# Editorial: Rising stars in nephrology 2024: illuminating the future of kidney health

**DOI:** 10.3389/fneph.2026.1985175

**Published:** 2026-09-16

**Authors:** Peter Kotanko

**Affiliations:** 1 Renal Research Institute, New York, NY, United States; 2 Division of Nephrology, Icahn School of Medicine at Mount Sinai, New York, NY, United States

**Keywords:** cardiovascular disease, chronic kidney disease, dialysis, kidney transplantation, pregnancy

## Introduction

Chronic kidney disease (CKD) now affects more than 850 million people worldwide, and its burden continues to grow even as the tools available to detect, stage, and treat it become more sophisticated. Meeting this challenge depends as much on the next generation of nephrologists and kidney scientists as on any single new drug or device.

This Research Topic was conceived to give early-career investigators a platform to present their original research, reviews, and clinical perspectives that push the field forward, and its eleven contributions span the full spectrum of nephrology: nephron physiology and disease staging, pregnancy-related electrolyte disorders, pharmacotherapy for progressive CKD, cardiovascular risk after transplantation, biomarkers of acute and chronic kidney injury, the burden of immunosuppressive treatment, and an instructive case at the intersection of infection and glomerular disease. Read together, they illustrate both the diversity of questions that early-career nephrologists are asking and several themes that recur across otherwise disparate work (**Figure 1**).

**Figure 1:**
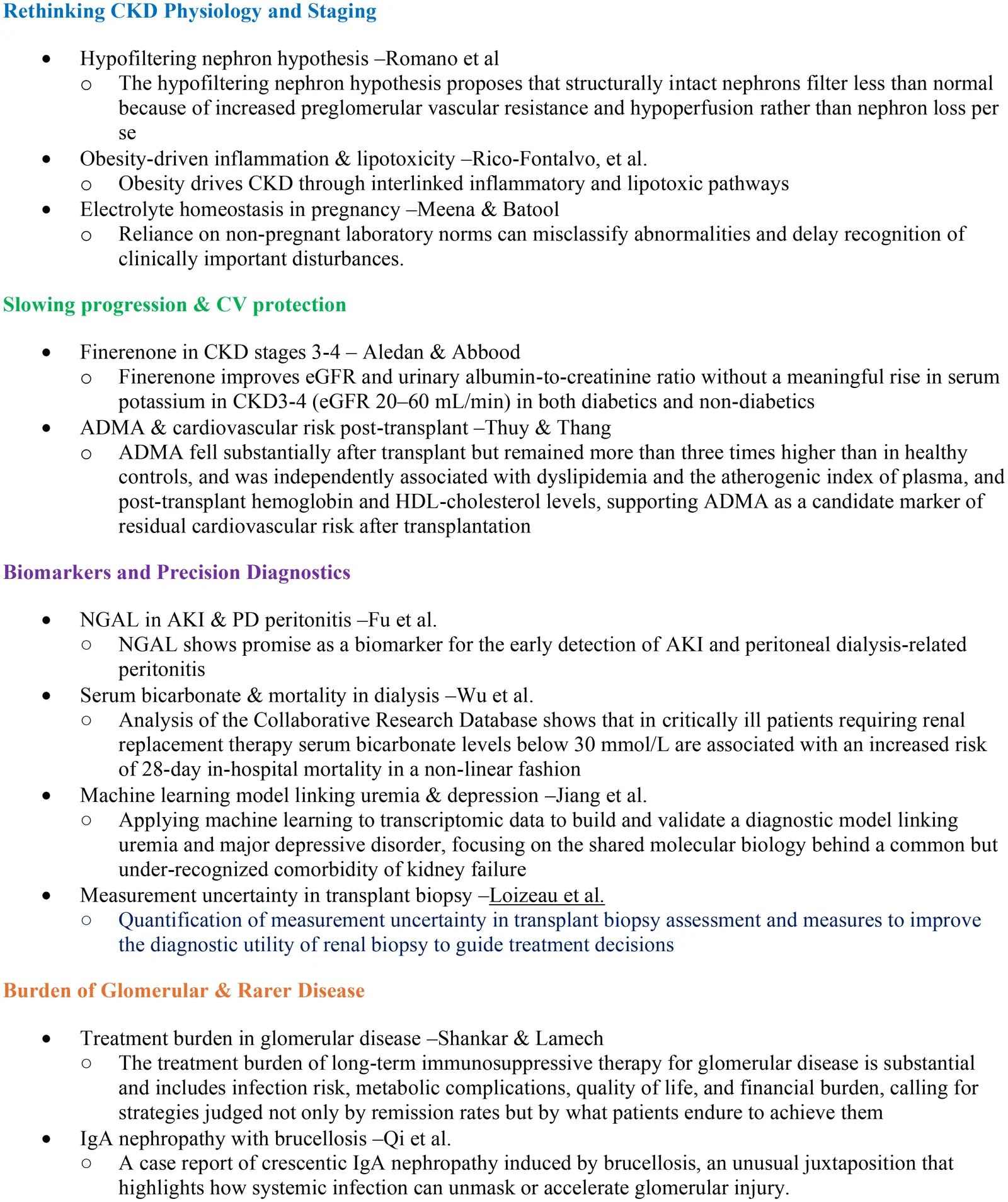
Rising stars in Nephrology 2024 contributing articles across four recurrent themes.

## Rethinking the physiology and staging of CKD

Several contributions challenge the assumption that CKD is a single, homogeneous entity once glomerular filtration rate (GFR) and albuminuria are known. Romano et al. revisit Bricker’s decades-old “intact nephron hypothesis,” which holds that surviving nephrons compensate for lost ones by hyperfiltering, and propose a complementary “hypofiltering nephron” model in which structurally intact nephrons filter less because of increased preglomerular vascular resistance and hypoperfusion rather than nephron loss per se. Because current KDIGO staging relies solely on estimated GFR and albuminuria categories, the authors argue that mechanistically distinct patients are too often treated identically, and propose that renal hemodynamic markers, such as the intrarenal Doppler-derived resistive index (RRI), could help personalize management. The authors claim that patients with CKD and hypofiltering residual nephrons should be treated differently than those with hyperfiltration of residual nephrons. This conceptual work is complemented by Rico-Fontalvo et al., whose mini review dissects how obesity drives CKD through interlinked inflammatory and lipotoxic pathways, reinforcing that metabolic phenotype, not eGFR alone, shapes disease trajectory. The authors discuss their proposed classification of obesity-related CKD.

Pregnancy offers a further illustration of why reliance on non-pregnant laboratory norms can misclassify abnormalities and delay recognition of clinically important disturbances. Meena and Batool synthesize how systemic vasodilation, neurohormonal activation, and maternal–fetal mineral exchange reset the normal ranges for osmoregulation, sodium-water homeostasis, potassium, magnesium, and calcium across gestation, providing a practical framework for distinguishing physiological adaptation from pathological disturbance in this understudied area of obstetric nephrology.

## Slowing progression and protecting the cardiovascular system

Two original research articles test therapies aimed at the outcomes that matter most to patients with CKD: kidney failure and cardiovascular disease. Aledan and Abbood report a prospective cohort study of finerenone in 180 patients with CKD stages 3–4 (eGFR 20–60 mL/min), including diabetic and non-diabetic participants and patients with eGFR as low as 20 mL/min, populations historically underrepresented in finerenone trials. Over one year, finerenone significantly improved eGFR and urinary albumin-to-creatinine ratio without a meaningful rise in serum potassium, extending the evidence base for this drug class to a broader, more advanced CKD population.

Cardiovascular risk after transplantation is the focus of Thuy and Thang‘s study of plasma asymmetric dimethylarginine (ADMA), an endogenous nitric oxide synthase inhibitor, in 75 patients before and six months after kidney transplantation. ADMA fell substantially after transplant but remained more than three times higher than in healthy controls, and was independently associated with dyslipidemia, the atherogenic index of plasma, and post-transplant hemoglobin and HDL-cholesterol levels, supporting ADMA as a candidate marker of residual cardiovascular risk even after otherwise successful transplantation.

## Biomarkers and precision diagnostics

A number of papers explores how biomarkers and computational methods can enhance and sharpen diagnosis. Fu et al. review neutrophil gelatinase-associated lipocalin (NGAL) as a biomarker for both acute kidney injury and peritoneal dialysis-related peritonitis, arguing for its potential as an early, non-invasive signal across two clinically distinct but mechanistically related complications. Wu et al. use the electronic Intensive Care Unit (eICU) Collaborative Research Database to examine the association between serum bicarbonate and 28-day in-hospital mortality in dialysis patients, adding evidence that acid–base status carries prognostic weight in critically ill patients on renal replacement therapy. Their research indicates that serum bicarbonate levels below 30 mmol/L are linked to an increased risk of death. Jiang et al. apply machine learning to transcriptomic data to build and validate a diagnostic model linking two chronic wasting diseases, uremia and major depressive disorder, and begin to map the shared molecular biology behind a common but under-recognized comorbidity of kidney failure. In the transplantation space, Loizeau et al. quantify measurement uncertainty in renal transplant biopsy assessment, a reminder that even the histopathologic “gold standard” for diagnosing rejection carries inter-observer variability that should inform treatment decisions.

## The burden of glomerular and rarer disease

Two contributions bring the discussion back to the patient. Shankar and Lamech catalog the treatment burden imposed by long-term immunosuppressive therapy for glomerular disease, weighing efficacy against infection risk, metabolic complications, and quality of life, financial burden, and calling for strategies judged not only by remission rates but by what patients endure to achieve them. Qi et al. contribute a case report of IgA nephropathy with partial crescentic lesions in a patient with brucellosis, an unusual juxtaposition that highlights how systemic infection can unmask or accelerate glomerular injury.

## Conclusion

These eleven articles span a notable range of topics, and that is the point of a Rising Stars collection: it captures the breadth of questions that early-career nephrologists are choosing to ask, from nephron-level physiology to population-scale database analyses, from pharmacologic trials to bioinformatic modeling of comorbidity. Several threads nonetheless recur — the limits of GFR and albuminuria as the sole means for staging CKD, the persistence of cardiovascular risk even after kidney replacement, the value of biomarkers that anticipate injury rather than merely confirm it, and the human cost of therapies used to control glomerular disease. We thank the authors, reviewers, and editors who contributed to this Research Topic, and we look forward to seeing how these emerging investigators continue to shape the future of kidney health.

